# A National Dental Association

**Published:** 1879-12

**Authors:** R. B. Winder

**Affiliations:** Baltimore, Md.


### ARTICLE III.

### *A National Dental Association.*

BY R. B. WINDER, M. D., D. D. S., BALTIMORE, MD.

(Read before the American Dental Convention at Saratoga, Aug. 12, 1879.)

*Mr. President and Gentlemen of the American Dental Convention*:—I have been requested to say something in regard to dental organizations, and I have to congratulate myself, not you, upon the opportunity thus afforded me of expressing my views in relation to these matters. * * *

We will not enter into a retrospective view of the means by which dentistry has arrived at its present stand-point of excellence. We are all familiar with the past history of our profession, and need not to have it repeated here.

We have to deal with the present and future, not the past. I think, however, we will all admit without discussion the premise, that Society organizations (both local and general) have had much to do with our advancement, and consequently we still need to use these levers to lift us to higher and better things. We thoroughly understand the importance of our State societies, and must therefore labor to keep them up to the highest stand-point. They are the fountains from which the streams of knowledge flow, the laboratories whence the main good comes. The general societies are more like a reservoir, intended to receive these blessings as they are poured in from these various rivulets; the grand emporium into which are carried all the valuable accumulations of labor and toil; the granaries where all the ripe fruits of a rich harvest are stored up as common property for future use and reference; a central library open to all; in the language of Dr. Atkinson, "a big camp-meeting," where all can meet, mingle and be happy.

It is useless, then, to discuss the question of the necessity and usefulness of either local, State, or general dental organizations. We feel quite certain that if these auxiliaries were swept away dentistry would soon stagnate. I shall not, therefore, in this paper say anything about our State organizations more than that we must have them, must cherish them, must nourish them, and that each State must, in its own wisdom, so arrange and govern its own society as to bring it up to the necessities of the case.

Regarding general associations, the times demand a national organization, which shall embrace in its membership all who are worthy, and know no North, South, East, or West, but whose purpose shall be the greatest good to the greatest number.

The advantages which a national organization would give to the whole profession of the United States are as legion, and we may with great propriety enumerate some of them here.

In the first place, I would say that if the United States are to hold among the different people of the earth the reputation in dentistry which has been so earnestly sought and assiduously labored for, it becomes absolutely necessary that we should have a representative and authoritative national organization, indorsed by all the different States, through their State societies, as an exponent of our progress, importance, standing, and culture. An organization of this sort would certainly carry much more weight and authority, both at home and abroad, than we could possibly expect from our present arrangements. It is the very first principle in military tactics—and I believe the same principle will apply in almost everything where there is any labor to be performed, any end to be obtained, or any difficulties or obstacles to be overcome and removed—that in order to *win* "forces must be concentrated, not scattered."

By this arrangement we could get all our materials together. All the best intellect and energies of the profession could, if necessary, be brought to bear upon one objective-point. Such an organization would be your base of supplies. You would have all your resources in hand, and could operate in any direction you might see fit. You would be clothed with national authority and power. We have much talent of one kind or another in the scattered ranks of our profession; it could in this way be brought together and utilized. Such an organization would of necessity wipe out all geographical lines and sectional prejudices (each district of country having the same guaranteed rights and privileges,) and the end would be the formation of one grand brotherhood, thoroughly united in everything that could tend to promote the present and future welfare and prosperity of dentistry, and in such a harmonious body of representative national men the highest perfection and attainment in dental art and science would be rapidly realized. This organization would also practically relieve us from much inconvenience and annoyance, from the fact that there are many of us members of the three large associations of the country who would willingly attend all their meetings, and who have for all of them the kindest feelings and best wishes, but who do not like to discriminate in favor of one or the other, and both time and money are wanting to effect a meeting with all. Surely something is due to the comfort and health of the dentist's family, and if his vacation and rest from labor involves the yearly attendance upon these meeeings, it becomes irksome. If the State societies would do the work which they should do, all we would need would be a national association where the members of the State societies might occasionally meet. This would not fall hard upon any one, and we could have zealous and earnest meetings, where we could commune, compare old with new, legislate if necessary, and separate and go back to our homes and report all the good that had been effected.

This organization would serve as a Mecca, from which each weary pilgrim might return blessed with the wealth of all the newly-discovered facts in art and science so lavishly bestowed upon all true worshipers at such a shrine.

There was a time when wandering tribes in good old patriarchial days carried with them their government and means of progress. This day has passed. The progress of civilization demands more than this. A country without a government or a head is only anarchy. The medical profession has its representative national organization. The scientists have theirs. Why should not we? We have nothing but praise to bestow upon the present dental organizations; they have fought a good fight and deserve all praise. They have accomplished much good, and deserve the respect and confidence of all. But this is no reason why we should not have a national organization. It strikes me that there can be no reasonable objection or opposition to this movement. Some people, however, will say, Have we not already a national organization? I would ask them, by way of reply, Can this possibly be so, when we have three general organizations,—The American Dental Convention, the American Dental Association, and the Southern (now National) Association? Does this look as if we had a National Association? You might say, We have several! But is there *one*,—*the* National? We have but one United States Senate and one House of Congress. We had two Senates and two Houses of Congress at one time. But was this an outgrowth of peace and harmony? There must be a head. The profession requires and the times demand this national organization. The American Dental Convention, which is the oldest general society in the country, and the Southern Association saw this two years ago, and passed the necessary resolutions towards effecting it. They made all sorts of overtures to the American Dental Association, and even went so far as to adjourn and meet at Niagara last year, so as to give the American Dental Association no possible or reasonable excuse for non-co-operation. The American Dental Association was invited the year before to meet with the American Dental Convention and the Southern Dental Association, but as I learn from good authority, declined, through a misconception of the object in view. So that, as the mountain would not come to Mahomet, Mahomet went to the mountain. But the American Dental Association still refused co-operation. We will not say one word against the American Dental Association. It has done much good, and it is the earnest hope and wish of the speaker that it will continue to live and flourish as long as its career is one of usefulnesss. The formation of a national organization does not militate against the interests of any other associations, and is not meant to destroy, but to save.

We will now briefly discuss the principles and general outlines upon which we can form such an organization. The first question that suggests itself to me is, How often and where shall we meet? I would suggest, in reply to this query, that we ought to meet annually,—one year in the North, one year in the South, one year in the East, one year in the West, and one year in the center, at Washington, our seat of government. I would have it meet annually in this way, in order that we may more effectually carry out the object of the organization,—the greatest good to the greatest number; because by swinging around the circle, we adapt our meetings to suit the necessities arising from the scattered condition of our rank and file, and bring the same high privileges within the easy reach of all. Our gain would be in having ultimately the whole profession with us, and our membership might be counted by thousands. Under this arrangement no one could afford to be left out in the cold. I would also suggest as a means of retaining this immense membership, and gradually utilizing it, that no dues be demanded from a member except when he was present at a meeting. With some of us the expense of dues is a very small matter, but there are others (and worthy members of the profession too,) who are not so fortunate. This arrangement would at last amount to the same thing, and place the onus of expense upon each geographical section of the country in turn. Every fifth year we should meet at the seat of government. Obviously we should do this in order to present the appearance of a national organization to the rest of the world, and in that year we might occasionally invite an international meeting. Unquestionably for this purpose our seat of government should be the meeting-place. But we have other reasons. It is to be hoped (if this organization should be a success) that it will gradually accumulate a library, scientific apparatus, and perhaps a museum, and we would certainly need some central place to store our archives. Again, it becomes important in its influence upon the government itself, if we ever hope to secure the appointment of dental surgeons to the army and navy of the United States,—a need which sooner or later must be supplied. For the reasons given, I would then earnestly advise that we should meet as has been suggested; that the geographical divisions of our territory should in the commencement be definitely agreed upon, so as to leave no room in the future for discussion or contention; and perhaps it would be well to ignore the idea of North, South, East, and West, and divide the country into geographical districts, making provision for territory that will in time become States, so that every part of the country should share the benefits.

In regard to the government of this national organization, I would earnestly recommend that the officers and executive committee should each year be chosen from the section or district of country in which the next meeting is to take place, and that the time and place of meeting be also left to the wishes and best judgment of the section or district in which it is to meet. Surely no one would desire to go to New Orleans in the heat of summer, or to Maine in the depth of winter. I think, too, that we should always have some one at Washington to take care of our archives, etc., who could act at the same time as corresponding secretary, so that the whole world might always know where and to whom to direct any communication to our national organization.

As the consummation, after this organization shall have gotten into good working order, I would have permanent sections, embracing the different subjects pertaining to the science and art of dentistry, formed of a limited number of life memberships, and selected upon merit alone,—scientific attainment and artistic skill. I would have these positions the posts of honor, and would offer them as a reward to the diligent and meritorious. Such a badge of distinction would be the highest award our profession could boast, and would stimulate young men to labor assiduously and persistently in some one avenue of science or art, hoping in the end to win and wear this honorable distinction. It would become the goal of the highest aspirations.

I have thus briefly given you a general outline of the principles upon which I think this grand national organization should be formed. But the organization itself will depend upon the report of your committees; the detail and minutiæ of government will have to be mapped out by these same committees, and I know and feel that it will take much time and careful consideration to bring everything into harmonious and proper working order. The question of membership is also to be considered and decided upon. If it can be so arranged, I think it most desirable to have our membership consist of delegates from State societies only. This would compel every State to form a State society or lose its representation; but I would prefer that all members of State societies should have the privilege of membership in this national body.

The Southern Dental Association met in July, in Augusta, Georgia, and has carried out in good faith its agreement with this convention to unite with it in forming a national organization, and has appointed the first Tuesday in September, 1880, to meet with you in the city of New York for the purpose of consummating this great scheme. The president, Dr. Patrick, of Charleston, South Carolina, has plenary powers to appoint the necessary working committees, etc. I learned on Monday that the American Dental Association has also appointed a committee (two of whom are present with us,—Prof. Pierce, of Philadelphia, and Dr.

McKellops, of St. Louis) to confer with our committees on this subject. We have a committee to appoint to aid in this great work, and I can only say, where so much is at stake, that I most sincerely hope that the members of our committee will be most carefully selected,—men of enlarged views, good judgement, intelligence, energy, and free from prejudices. All these qualifications will be necessary for this Herculean task.

I thank you for your very kind attention, and most sincerely hope that our fondest expectations in regard to this great undertaking may be fully realized at our ratification meeting on the first Tuesday in September, 1880, in the city of New York.—*Dental Cosmos.*

